# Effect of Bovine Milk Fat Globule Membrane and Lactoferrin in Infant Formula on Gut Microbiome and Metabolome at 4 Months of Age

**DOI:** 10.1093/cdn/nzab027

**Published:** 2021-04-02

**Authors:** Maciej Chichlowski, Nicholas Bokulich, Cheryl L Harris, Jennifer L Wampler, Fei Li, Carol Lynn Berseth, Colin Rudolph, Steven S Wu

**Affiliations:** Medical and Scientific Affairs, Reckitt|Mead Johnson Nutrition Institute, Evansville, IN, USA; Laboratory of Food Systems Biotechnology, Institute of Food, Nutrition, and Health, ETH Zürich, Zurich, Switzerland; Medical and Scientific Affairs, Reckitt|Mead Johnson Nutrition Institute, Evansville, IN, USA; Medical and Scientific Affairs, Reckitt|Mead Johnson Nutrition Institute, Evansville, IN, USA; Departments of Developmental and Behavioral Pediatrics & Child Primary Care, MOE-Shanghai Key Lab for Children's Environmental Health, Xinhua Hospital Affiliated to Shanghai Jiaotong University School of Medicine, Shanghai, China; Medical and Scientific Affairs, Reckitt|Mead Johnson Nutrition Institute, Evansville, IN, USA; Medical and Scientific Affairs, Reckitt|Mead Johnson Nutrition Institute, Evansville, IN, USA; Department of Pediatrics, University of California, San Francisco, San Francisco, CA, USA; Medical and Scientific Affairs, Reckitt|Mead Johnson Nutrition Institute, Evansville, IN, USA; Division of Pediatric Gastroenterology, Indiana University School of Medicine, Indianapolis, IN, USA

**Keywords:** milk fat globule membrane (MFGM), lactoferrin, infant microbiome, metabolome, formula feeding

## Abstract

**Background:**

Milk fat globule membrane (MFGM) and lactoferrin (LF) are human-milk bioactive components demonstrated to support gastrointestinal and immune development. Significantly fewer diarrhea and respiratory-associated adverse events through 18 mo of age were previously reported in healthy term infants fed a cow-milk–based infant formula with an added source of bovine MFGM and bovine LF through 12 mo of age.

**Objectives:**

The aim was to compare microbiota and metabolite profiles in a subset of study participants.

**Methods:**

Stool samples were collected at baseline (10–14 d of age) and day 120. Bacterial community profiling was performed via 16S rRNA gene sequencing and alpha and beta diversity were analyzed (QIIME 2). Differentially abundant taxa were determined using linear discriminant analysis effect size (LefSE) and visualized (Metacoder). Untargeted stool metabolites were analyzed (HPLC/MS) and expressed as the fold-change between group means (control to MFGM+LF ratio).

**Results:**

Alpha diversity increased significantly in both groups from baseline to 4 mo. Subtle group differences in beta diversity were demonstrated at 4 mo (Jaccard distance; *R*^2^ = 0.01, *P* = 0.042). Specifically, *Bacteroides uniformis* and *Bacteroides plebeius* were more abundant in the MFGM+LF group at 4 mo. Metabolite profile differences for MFGM+LF versus control included lower fecal medium-chain fatty acids, deoxycarnitine, and glycochenodeoxycholate, and some higher fecal carbohydrates and steroids (*P *< 0.05). After applying multiple test correction, the differences in stool metabolomics were not significant.

**Conclusions:**

Addition of bovine MFGM and LF in infant formula was associated with subtle differences in stool microbiome and metabolome by 4 mo of age, including increased prevalence of *Bacteroides* species. Stool metabolite profiles may be consistent with altered microbial metabolism. This trial was registered at https://clinicaltrials.gov as NCT02274883.

## Teaser

This study assessed the impact of infant formula with added bovine MFGM and lactoferrin on stool microbiome and metabolome at 4 months of age.

## Introduction

Maturation of the infant immune system and gastrointestinal (GI) tract, including dynamic development of the gut microbiome, establishes the foundation for long-term metabolic and immune health (1). Diet is central to infant growth, health, and intestinal development, and human milk (or its substitution) is recognized as the most significant factor shaping the microbiome (2). Ongoing investigation into human milk has provided new information on functional components, such as the milk fat globule membrane (MFGM). MFGM envelopes milk-fat globules, facilitates their secretion from the mammary gland, and is rich in both nonglycosylated and glycosylated bioactive proteins and lipids (3) and includes components with antimicrobial effects including gangliosides (4), oligosaccharides (5), and the glycoproteins butyrophilin, lactadherin, and mucins (6). Previous research suggests that human MFGM components may affect bacterial attachment, colonization, clearance, and metabolism in the oral cavity and the gut (7).

Due to homology between human and bovine MFGM, the incorporation of bovine MFGM (bMFGM) in infant formula (IF) may better approximate the composition of complex human-milk lipids (8, 9). Animal studies have demonstrated the impact of bMFGM on microbiome outcomes. In piglet models, animals who received formula with added bMFGM displayed significant group differences in ascending colon and stool microbiota (10), as well as higher stool Proteobacteria and reduced Firmicutes (11). In rat pups receiving formula with added bMFGM, richness and evenness of gut microbial species increased compared with a control formula with added vegetable fat, and the abundance of Firmicutes and Proteobacteria were more similar to a group receiving dam's milk (3). In the same study, Lactobacilli were most abundant in dam-reared pups, present in the bMFGM group, and not detected in the control group. In stress-induced juvenile rats receiving a diet with bMFGM, lactoferrin (LF), and prebiotics, improvements in sleep outcomes corresponded to increased growth of select beneficial taxa and protection against the stress-induced decrease in alpha diversity (12). In infants fed formula with added bMFGM, moderate effects on overall microbiome structure (oral and stool) were demonstrated (13) and the common middle-ear pathogen *Moraxella catarrhalis* was less prevalent in oral swabs at 4 mo of age (vs. infants receiving formula with no added bMFGM) (14).

In healthy term infants randomly assigned to a cow-milk-based IF (MFGM+LF) with added bMFGM (Lacprodan^®^ MFGM-10; Arla Foods Ingredients) and bovine lactoferrin (bLF; 0.6 g/L; FrieslandCampina DMV) compared with a similar control formula (no added bMFGM) through 12 mo, an accelerated neurodevelopmental profile by 12 mo of age and effects on language by 18 mo of age was reported (15). Added bMFGM and bLF in formula was also associated with significantly fewer episodes of respiratory adverse events and diarrhea through 18 mo of age, in line with a prior report of decreased diarrhea in infants receiving MFGM (16). Because the effects of early nutrition on the GI microbiome may potentially contribute to overall health and developmental outcomes, stool samples were collected in a subset of study participants at 4 mo of age, with the goal of comparing GI microbiome outcomes and metabolite profiles.

## Methods

### Participants and study design

In this double-blind, controlled, prospective study (trial registration: https://clinicaltrials.gov/ct2/show/NCT02274883), healthy term infants (born at 37–42 wk of gestation) were randomly assigned to receive the following: a cow-milk-based IF or a similar IF that had added bMFGM and LF from baseline (10–14 d of age) through 365 d of age. Mothers who had decided to exclusively provide IF were screened for study eligibility and eligible infants had received IF feeding for ≥3 d prior to randomization. As described previously, the primary outcome was the Bayley Scales of Infant Development, 3rd edition (Bayley-III), composite score at day 365 (15). Stool samples were collected in a subset of participants to evaluate stool microbiota at baseline and day 120 (MFGM+LF: *n* = 27; control: *n* = 35) and metabolite profiles at day 120 (MFGM+LF: *n* = 26; control: *n* = 33). Additional criteria for inclusion in the subset included the following: vaginal birth, no antibiotic/corticosteroid treatment or prebiotic/probiotic supplements, and access to a home freezer. For each analysis, a stool sample of approximately 5 mL was collected at home and stored in the home freezer. Samples were de-identified at the time of collection. After delivery to the study site, all samples were maintained at −80°C until processing and analysis.

The research protocol and informed consent forms observing the Declaration of Helsinki (including October 1996 amendment) were approved by the Shanghai Nutrition Academy Medical Ethical Committee.

### 16S rRNA sequencing and analysis

Genomic DNA was extracted in duplicate from stool samples (PowerSoil DNA Isolation Kit; MoBio Laboratories, Inc.). The 16S rRNA gene-specific primer pair 515F (5″-GTGCCAGCMGCCGCGGTAA-3″) and 806R (5″-GGACTACHVGGGTWTCTAAT-3″) (17) was selected to amplify the V4 region of 16S rDNA. Sample preparation and high-throughput sequencing of the qualified libraries were conducted (BGI Tech Solutions Co., Ltd., Wuhan, China) using the MiSeq NGS platform (Illumina, Inc.) and paired-end 2 × 250-bp (PE250) sequencing strategy. Marker-gene sequence data were processed and analyzed using the plugin-based microbiome bioinformatics framework QIIME 2 version 2019.4 (18). DADA2 (19) was used (via the q2-dada2 QIIME 2 plugin) to filter and correct sequencing errors, remove PhiX and chimeric reads, and join the paired-end sequence reads into amplicon sequence variants (ASVs; enables distinguishing between sequences on a single-nucleotide level). To infer a phylogenetic tree, ASVs were inserted into Greengenes version 13_8 reference phylogeny (20) using the q2-fragment-insertion plugin (21). ASVs were taxonomically classified using q2-feature-classifier (22) with the classify-sklearn method against the Greengenes 16S rRNA reference database 13_8 release (20).

Alpha diversity (within-sample diversity) analyses were performed using the QIIME 2 q2-diversity plugin to measure richness (as observed ASVs). To quantify the dissimilarity between microbial samples by study time point and group, we examined multiple beta diversity metrics in QIIME 2: unweighted UniFrac (a phylogenetic, qualitative metric), weighted Unifrac (phylogenetic, abundance-weighted), Bray-Curtis dissimilarity (nonphylogenetic, abundance-weighted), and Jaccard distance (nonphylogenetic, qualitative) (23). Feature tables were evenly subsampled at 27,000 sequences per sample prior to alpha and beta diversity analyses. Two-factor ANOVA tests and Wilcoxon signed-rank tests were performed using the q2-longitudinal plugin (24) to test whether alpha diversity estimates differed between study time point and group. Pairwise distances were used to construct principal coordinates analysis plots and perform 2-way permutational multivariate ANOVA (PERMANOVA) tests (25) [as implemented in the Adonis method in the vegan R package (26), wrapped via the q2-diversity plugin] to test whether beta diversity estimates partitioned by study time point and group.

Differential heatmaps of taxonomic compositions in each sample (by study time point and group) were visualized using Metacoder (27). Analysis of composition of microbiomes (ANCOM) (28), linear discriminant analysis effect size (LefSE) (29), and Wilcoxon rank-sum tests (implemented in the Metacoder package) were also used to test differential ASV abundance by study time point and group.

### Stool metabolite analysis

A minimum of 100 mg of stool sample was needed to prepare and complete metabolite analysis. Briefly, samples were prepared using a methanol extraction to remove the protein fraction while allowing maximum recovery of small molecules and subsequently prepared for either LC/MS or GC/MS analysis. Metabolomic profiling was performed by Metabolon, Inc. (Research Triangle Park, NC), as previously described (30, 31). Differential abundance of metabolites between groups was identified using Kruskal-Wallis H-tests as implemented in Scipy (32) followed by Benjamini-Hochberg correction for multiple testing as implemented in Statsmodels (33).

### Correlations between bacteria and metabolites

The QIIME 2 plugin version of mmvec (https://github.com/biocore/mmvec) was used to predict associations between stool bacterial ASVs and stool metabolites using the microbe-metabolite vectors (mmvec) method (34). This method utilizes neural networks to predict microbe-metabolite log-conditional probabilities (i.e., the probability of observing a metabolite at a certain frequency as a function of microbial composition). Log-conditional probabilities between each microbe-metabolite pair were calculated using mmvec with default settings, ranked by score, and top scores were visualized with a heatmap.

## Results

### Age and feeding effects on stool bacterial community diversity

**FIGURE 1:**
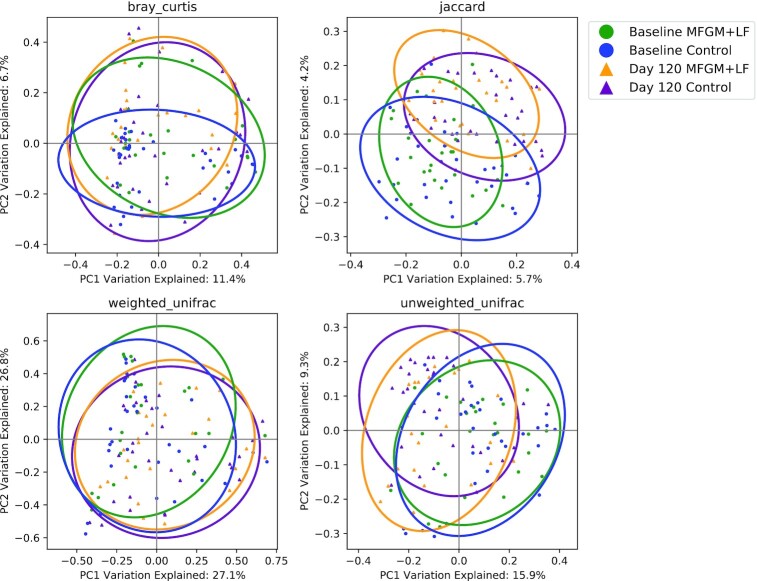
Degree of similarity between each sample at each study time point using beta diversity principal coordinate analysis plots. Each point represents a single sample, and the distance between each sample pair indicates the degree of dissimilarity of bacterial communities in those samples (proximal samples are more similar). Ellipses indicate the 95% CI. LF, lactoferrin; MFGM, milk fat globule membrane; PC, principal coordinate.

Observed species (richness, representing the count of unique ASV observed in each sample) were significantly different between baseline and day 120 (Kruskal Wallis, *P *< 0.001; data not shown). No group differences in bacterial composition or species richness (ASVs) from baseline to day 120 were detected (paired Wilcoxon tests; **Supplemental Figures 1** and **2**). Age exerted the strongest effect on bacterial community composition by all measures of beta diversity (**Table 1**), although age explained only a small degree of variance in beta diversity (max *R^2^* = 0.06 with unweighted UniFrac). Using Jaccard distance (*R^2^* = 0.01, *P* = 0.042), low-abundance, phylogenetically related species differed between groups (**Figure 1**). No significant differences between study feeding groups at baseline or day 120 were detected by Bray-Curtis and Unifrac (weighted or unweighted) analysis.

**TABLE 1:** PERMANOVA Beta diversity difference tests indicate that age, but not feeding group, is associated with bacterial community differences^1^

	*df*	Unweighted UniFrac, *R^2^*	*P*	Weighted UniFrac, *R^2^*	*P*	Bray-Curtis, *R*^2^	*P*	Jaccard, *R*^2^	*P*
Age	1	0.06	0.001	0.04	0.002	0.03	0.001	0.03	0.001
Group	1	0.01	0.427	0.00	0.769	0.01	0.201	0.01	0.042
Age:group	1	0.01	0.773	0.00	0.958	0.01	0.750	0.01	0.678
Residuals	114	0.93		0.95		0.95		0.95	

^1^PERMANOVA, permutational multivariate ANOVA.

### Relative microbial abundance

**FIGURE 2:**
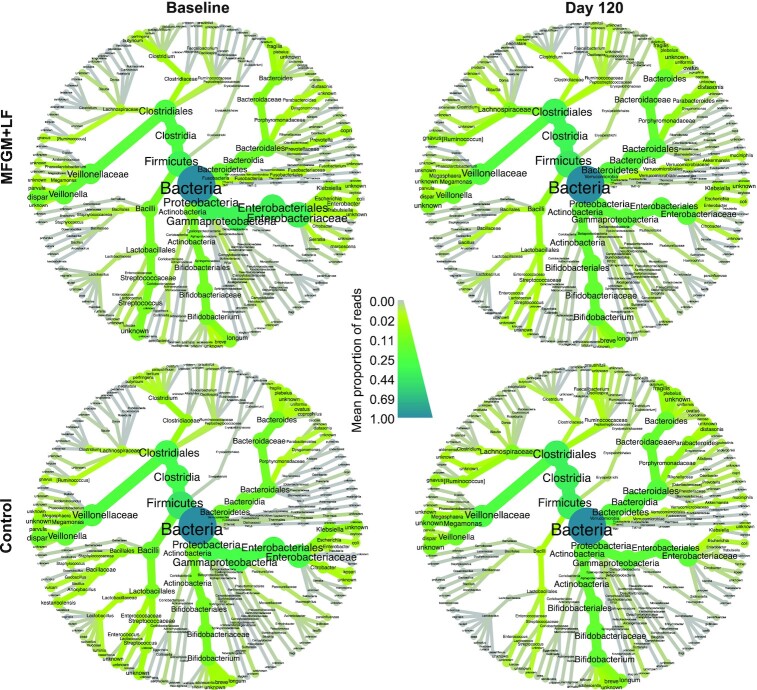
Differential heat trees display the mean proportion of bacterial components by study time point and group. Nodes represent each taxonomic rank from kingdom (bacteria, center) to species (tips of each branch). Node and edge (branch) width indicates the mean proportion of that taxon in samples belonging to that group. Size of nodes corresponds to the number of taxa and color intensity corresponds to proportions relative to bacterial samples overall. Only species detected at ≥0.01 mean proportion are displayed.

Analysis of core microbes for all samples revealed a high prevalence of select bacterial taxa at the species level by heatmap visualization (**Supplemental Figure 3**). Both study time points and groups were characterized by diverse bacterial communities dominated by various *Clostridiales* (principally *Veillonella* spp., *Megasphaera* spp., *Lachnospiraceae* spp., *Ruminococcus gnavus*, and *Faecalibacterium prausnitzii*), *Bacteroides* spp., *Parabacteroides* spp., *Bifidobacterium* spp. (primarily *B. longum*), *Enterobacteriaceae* (primarily *Klebsiella* spp. and *Escherichia coli*), and *Lactobacillales* (*Streptococcus, Enterococcus, Lactobacillus*) (**Figure 2**).

**FIGURE 3:**
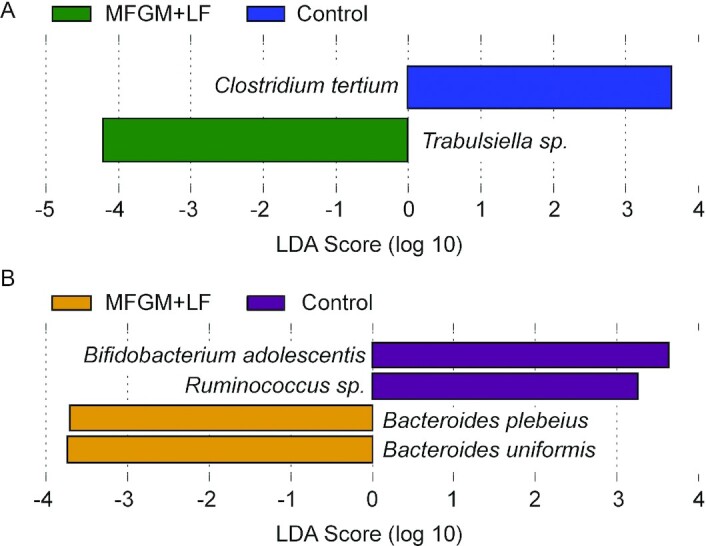
Identification of bacterial species that differentiated MFGM+LF vs. control feeding groups by LDA effect size (*P *< 0.05) at baseline (A) and day 120 (B). LDA, linear discriminant analysis; LF, lactoferrin; MFGM, milk fat globule membrane.

Differential stool bacterial species abundance between study feeding groups was tested using ANCOM, LEfSe, and MetaCoder (Wilcoxon rank-sum tests). These 3 tests were used to evaluate results using tests with different degrees of sensitivity and precision; the most conservative test, ANCOM, detected no significant features at either time point. LefSE and Wilcoxon tests were subsequently compared to assess what features may differ between feeding groups at baseline and day 120. No significant group differences in species were detected by ANCOM (data not shown), whereas LEfSe has shown several significant differences (**Figure 3**). At baseline, *Trabulsiella* sp. was more abundant in the MFGM+LF group and *Clostridium tertium* was more abundant in the control group. At day 120, *Bacteroides plebeius* and *Bacteroides uniformis* were more abundant in the MFGM+LF group and *Bifidobacterium adolescentis* and *Ruminococcus* sp. were more abundant in the control group.

### Effects of study feeding on stool metabolite abundance

**FIGURE 4:**
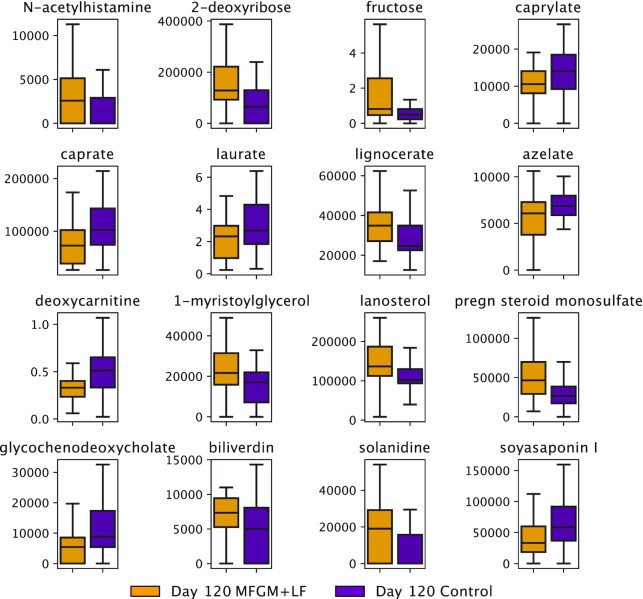
Differentially abundant metabolites distinguish MFGM+LF vs. control groups by day 120. Box and whisker plots show quartile values; center lines are medians, boxes are first and third quartiles, and whiskers are 95% CIs. Outliers are excluded for clarity. Only significant metabolites are shown (Kruskal-Wallis, *P* < 0.05). LF, lactoferrin; MFGM, milk fat globule membrane.

Untargeted metabolite analysis was performed on day 120 samples. Of the 569 total biochemicals detected (418 named and 151 unnamed), 20 were differentially abundant between feeding groups (Kruskal-Wallis, *P* < 0.05; **Figure 4**). Metabolites at higher abundance in the MFGM+LF group included carbohydrates (fructose, 2-deoxyibose), steroids (lanosterol, pregn steroid monosulfate), lipids (lignocerate, 1-myristoylglycerol), N-acetylhistamine, and biliverdin. Metabolites at higher abundance in the control group samples included the medium-chain fatty acids (caprylate, caprate, and laurate), the bile acid glycochenodeoxycholate, and deoxycarnitine. Otherwise, the fecal metabolic profiles were generally similar between feeding groups. However, after applying Benjamini-Hochberg multiple test correction, the differences were not significant.

**FIGURE 5:**
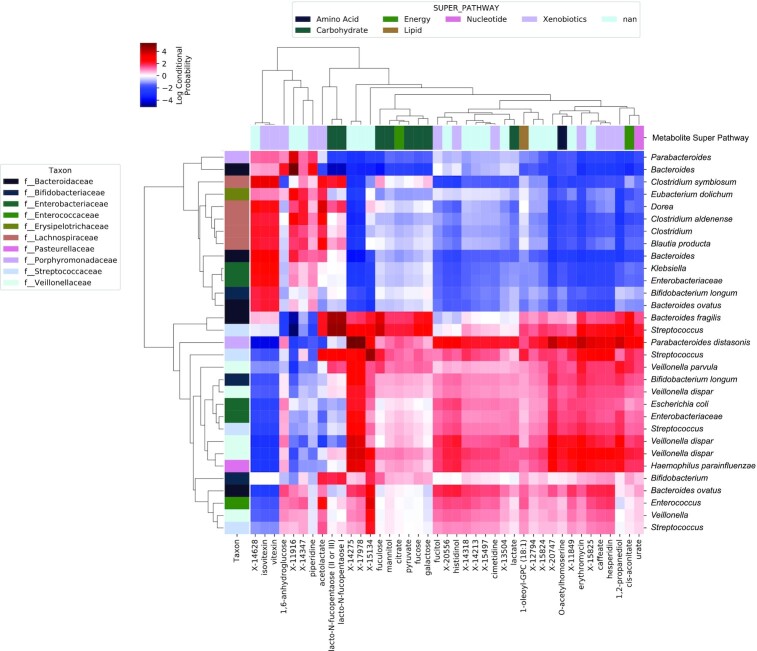
Microbe-metabolite vector analysis identifies co-occurrence probabilities between microbial ASVs and metabolites. Each row represents an individual bacterial ASV, labeled by its species ID, and each column represents an individual metabolite. The intersection of each row/column indicates their co-occurrence probability as an LCP. High LCPs (red) indicate strong co-occurrence probabilities between those features, and low LCPs (blue) indicate negative or null correlations. Margin vectors are colored according to bacterial family affiliation of taxa displayed along the *y*-axis and metabolite super family classification of metabolites displayed along the *x*-axis. Only the most significant correlations (features with at least 1 LCP >1.5) are shown. ASV, amplicon sequence variant; LCP, log conditional probability score.

Microbe-metabolite vectors (mmvec) were used to identify correlations between microbial taxa and stool metabolites at day 120. Results indicated several co-occurrence clusters: *1*) a cluster of *Lachnospiraceae*, *Enterobacteriaceae*, *Bacteroides* spp., *Parabacteroides*, *Eubacterium dolichum*, and *Bifidobacterium longum* associated with several xenobiotics (vitexin, isovitexin, piperidine, 1,6-anhydroglucose, acetolactate), lacto-N-fucopentaoses, and several unidentified metabolites (**Figure 5**). A second cluster, consisting of *Veillonella* spp., *Bacteroides* spp., *Streptococcus*, *Parabacteroides distasonis*, *Bifidobacterium* spp., *Enterobacteriaceae*, *Enterococcus*, and *Haemophilus parainfluenzae*, were associated with urate, *cis*-aconitate, O-acetylhomoserine, 1-oleoyl-GPC, lactate, citrate, and several carbohydrates, xenobiotics, and unidentified metabolites (Figure 5). None of these metabolites were among the differentially abundant metabolites described above.

## Discussion

The aim of the present analysis was to examine the impact of bMFGM and bLF in IF on stool microbiome and metabolome. We previously demonstrated improvements in neurodevelopmental testing outcomes and reductions in respiratory adverse events and diarrhea in infants receiving added bMFGM and bLF in formula (compared with a control) through 12 mo of age (15). In a subset of these infants (*n *= 62), we utilized V4–16S rRNA gene sequencing to characterize fecal bacterial community development during the first 4 mo of life.

Bacterial microbiota diversity can be quantified as the amount of variation in the microbial community within a sample (alpha diversity) or between samples (beta diversity). In many circumstances, diversity is considered an indicator of community stability and resilience—that is, higher diversity is associated with more stable community and lower diversity relates to less stable community. However, the microbiota of infants receiving human milk is more stable over time and is characterized by lower alpha diversity compared with infants receiving IF (35). Results in the current study demonstrated no significant effects of the diet on alpha diversity at the bacterial family level, suggesting that species richness increased with infant age but study feeding had less impact on the rate of change. Our results agree with a previous clinical study that reported a minimal effect on stool bacterial microbiota diversity in infants receiving bMFGM in IF (36). Timby et al. (37) showed that *Moraxella catarrhalis* was significantly lowered in oral microbiota in infants fed formula with bMFGM. *M. catarrhalis* is a common pathogen in otitis media, which was significantly less abundant in infants receiving added bMFGM in formula (14). Our analysis has shown that only low-abundance, phylogenetically related species differed between groups, explaining a very low degree of variation in beta diversity.

In the current study, LefSE analysis demonstrated an increase in 2 species of Bacteroides in the MFGM+LF group at day 120: *B. uniformis* and *B. plebeius*. Previously, stool *B. uniformis* has been reported as being higher in breastfed infants (38) and the authors further speculated that *B. uniformis* uses human-milk oligosaccharides (HMOs) to thrive in the infant gut from the early postnatal stage. This is supported by a separate report of a glycan utilization system identified in infant gut-associated *B. uniformis* that specifically facilitates the metabolism of mother's milk (39). Evidence for the effects of *B. uniformis* includes the finding that the strain *B. uniformis* CECT 7771, present in human milk, ameliorates metabolic and immune dysfunctions associated with obesity in a mouse model (40, 41). Since both bovine and human MFGM contain glycolipids and glycoproteins, these may provide glycan components utilizable by *B. uniformis*. Furthermore, it has been observed that the genus Bacteroides as a whole are one of the taxa most affected by delivery mode; lower levels were reported in infants born by cesarean delivery (42) or prematurely (43), whereas infants born vaginally have higher amounts of Bacteroides throughout the first year of life (42, 44–47). This suggests that Bacteroides, among other taxa, could be considered as a marker of relative eubiosis in infancy.

Other microbiome changes have been associated with components of bMFGM. Infants receiving a formula with added bovine gangliosides, a component of bMFGM, displayed an increase in Bifidobacteria and reduction in fecal *E. coli* compared with infants receiving a standard formula (48). In the present study, differences between the feeding groups were also observed at baseline. Because infants in the MFGM+LF group began receiving added bMFGM, bLF, and a blend of polydextrose and galacto-oligosaccharides in formula within the first few weeks of life, it is possible that an influence of even a few days of feeding early in life had an impact on the bacterial microbiota results.

Metabolomics analysis in the current study detected no significant differences between MFGM+LF and control groups. However, several important trends that are likely related to microbial metabolism were observed. For example, in the current study, stool lactate increased by day 120 in infants receiving added bMFGM and bLF in IF. Lactate is produced by Bifidobacteria, Bacteroides, Enterococci, and Streptococci (49), which are also primary colonizers of the infant gut and often associated with higher stool lactate in breastfed infants (50).

In a previous study of infants receiving added bMFGM in IF, lower fecal concentrations of amino acids (e.g., isoleucine, glutamate, phenylalanine, tyrosine, valine, and glycine) and 2-hydroxyisovalerate (branched-chain amino acid degradation product), cadaverine (lysine breakdown product), and 4-hydroxyphenyllactate (tyrosine breakdown product) were observed (vs. infants receiving control formula) (36). The present study also demonstrated a modest impact on infant stool metabolome, with added bMFGM and bLF in formula associated with lower medium-chain fatty acids and deoxycarnitine, although another amino acid metabolite, N-acetylhistamine, was elevated relative to the control group. Following Benjamini-Hochberg multiple test correction, none of the stool metabolites were significant despite differential abundance in MFGM+LF versus control groups.

The correlation analysis has shown several co-occurrence clusters (Supplemental Figure 3). None of these metabolites are among the differentially abundant metabolites, suggesting that the co-occurrence patterns here likely reflect the “core microbiome” and “core metabolome” in these subjects, rather than processes impacted by study feeding. We also acknowledge that the low sample to observation ratio limits our statistical power, and the uncorrected *P* values may contain many false positives. Given these limitations, we used this analysis as an exploratory approach for assessing how added bMFGM and bLF in IF may impact the stool metabolome. In addition, the analysis would have benefited from stool samples collected from a larger number of participants as well as samples from infants delivered by cesarean section.

In summary, our study has shown modest effects of supplementing IF with bMFGM and bLF on stool microbiome and metabolites, including (subtle) group differences in beta diversity (Jaccard distance) at 4 mo of age and higher abundance of select Bacteroides species. While such changes may contribute to the beneficial GI outcomes previously shown for formula with added bMFGM and bLF, this modest alteration of the gut microbiome is unlikely to represent the sole or primary driving factor, and additional mechanisms remain to be explored.

## Supplementary Material

nzab027_Supplemental_Files

## Data Availability

Data described in the manuscript will be made available upon request. The authors and study sponsor encourage and support the responsible and ethical sharing of data from clinical trials. De-identified participant data from the final research dataset used in the published manuscript may only be shared under the terms of a Data Use Agreement. Requests may be directed to steven.wu2@rb.com.

## Notes

This study was funded by the study sponsor, Reckitt|Mead Johnson Nutrition Institute (Evansville, IN, USA).

Author disclosures: FL’s institution was provided funding in order to implement the study protocol and study site coordination. NB was provided funds to prepare bioinformatics and statistical analyses. MC, CR, JLW, SSW and CLH are employees of Reckitt|Mead Johnson Nutrition Institute. CLB was previously employed by Reckitt|Mead Johnson Nutrition Institute and is currently employed at Brightseed, San Francisco, CA..

Supplemental Methods and Supplemental Figures 1–3 are available from the “Supplementary data” link in the online posting of the article and from the same link in the online table of contents at https://academic.oup.com/cdn/.

Abbreviations used: ANCOM, analysis of composition of microbiomes; ASV, amplicon sequence variant; bLF, bovine lactoferrin; bMFGM, bovine milk fat globule membrane; GI, gastrointestinal; GOS, galactooligosaccharide; IF, infant formula; LefSE, Linear discriminant analysis effect size; LF, lactoferrin; MFGM, milk fat globule membrane.
