# Supplementary material for: Effect of Bovine Milk Fat Globule Membrane and Lactoferrin in Infant Formula on Gut Microbiome and Metabolome at 4 Months of Age
Source: Curr Dev Nutr. 2021 Apr 2;5(5):nzab027. doi: 10.1093/cdn/nzab027 (PMC8105244; doi:10.1093/cdn/nzab027)
Supplement: nzab027_Supplemental_Files [file nzab027_supplemental_files.zip › Supplemental_Methods.docx]

*Stool Metabolite Analysis*

Stool metabolite concentrations were measured using untargeted LC/MS and GC/MS platforms (Metabolon Inc., Durham, NC USA). Briefly, samples were prepared using a methanol extraction to remove the protein fraction while allowing maximum recovery of small molecules and subsequently prepared for either LC/MS or GC/MS analysis. The LC/MS analysis was based on a Waters ACQUITY ultra-performance liquid chromatography (UPLC) and a Thermo Scientific Q-Exactive high resolution/accurate mass spectrometer interfaced with a heated electrospray ionization (HESI-II) source and Orbitrap mass analyzer operated at 35,000 mass resolution. The sample extract was dried then reconstituted in acidic or basic LC-compatible solvents, each of which contained 8 or more injection standards at fixed concentrations to ensure injection and chromatographic consistency. One aliquot was analyzed using acidic positive ion optimized conditions and the other using basic negative ion optimized conditions in two independent injections using separate dedicated columns (Waters UPLC BEH C18-2.1x100 mm, 1.7 µm). Extracts reconstituted in acidic conditions were gradient eluted from a C18 column using water and methanol containing 0.1% formic acid. The basic extracts were similarly eluted from C18 using methanol and water with 6.5mM ammonium bicarbonate. The third aliquot was analyzed via negative ionization following elution from a HILIC column (Waters UPLC BEH Amide 2.1x150 mm, 1.7 µm) using a gradient consisting of water and acetonitrile with 10mM ammonium formate. The MS analysis alternated between MS and data-dependent MS2 scans using dynamic exclusion (scan range 80-1000 m/z). The samples for analysis by GC-MS dried under vacuum for a minimum of 18 h prior to being derivatized under dried nitrogen using bistrimethyl-silyltrifluoroacetamide. Derivatized samples were separated on a 5% diphenyl/9 dimethyl polysiloxane fused silica column (20m x 0.18mm ID; 0.18 μm film thickness) with helium as carrier gas and a temperature ramp from 60° to 340°C in a 17.5 min period. Samples were analyzed on a Thermo-Finnigan Trace DSQ fast-scanning single-quadrupole mass spectrometer (scan range, 50-750 m/z) using electron impact ionization (EI) and operated at unit mass resolving power. Data were expressed as fold difference between group means and analyzed by Welch’s two-sample t-test to identify biochemical differences between treatment groups.
