# Supplementary material for: Effect of Bovine Milk Fat Globule Membrane and Lactoferrin in Infant Formula on Gut Microbiome and Metabolome at 4 Months of Age
Source: Curr Dev Nutr. 2021 Apr 2;5(5):nzab027. doi: 10.1093/cdn/nzab027 (PMC8105244; doi:10.1093/cdn/nzab027)

Suppl. Figure 1. Observed ASVs detected by study time point and group. Age has a significant effect (ANOVA P < 0.05) but not group (P > 0.05). Box and whisker plots show quartile values; center lines are medians, boxes are first and third quartiles, and whiskers are 95% CI. Outliers are excluded for clarity.


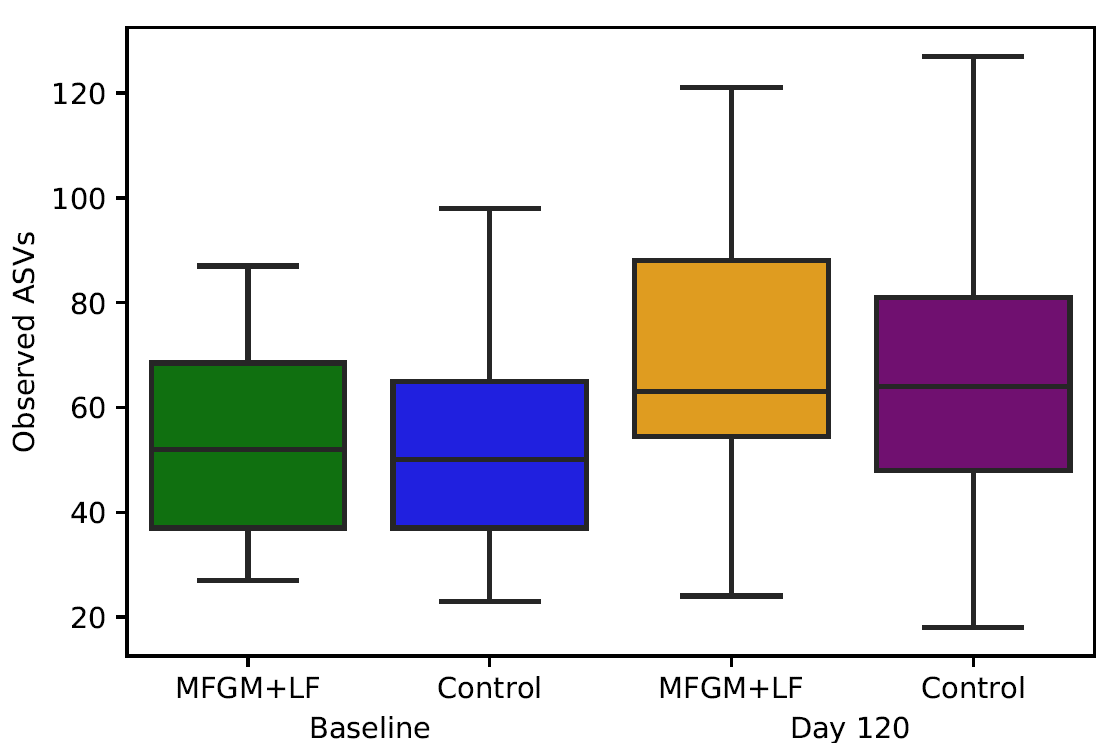


Suppl. Figure 2. Random Forest classification identifies bacterial ASVs that differentiate children’s ages. This heatmap shows the top 50 most predictive ASVs (labeled by taxonomy) and their normalized relative abundances in each sample. x-axis margin colors indicate time point. Samples are hierarchically clustered by UPGMA of correlation distance (in other words, samples with similar abundances of these ASVs cluster together, and ASVs are clustered by co-occurrence patterns).


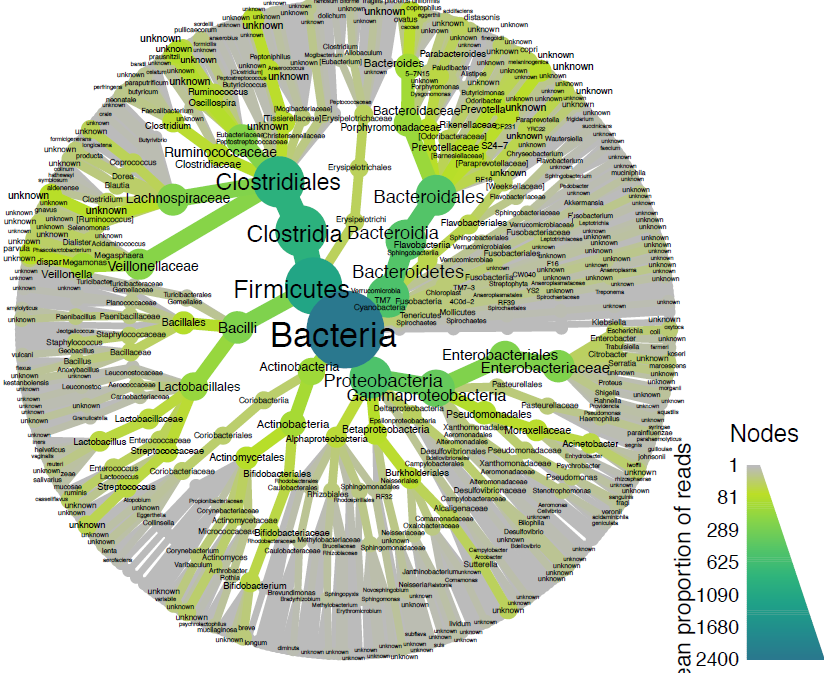


Suppl. Figure 3. Heat tree displaying the mean proportion of bacterial components across all samples. Nodes represent each taxonomic rank from kingdom (Bacteria, center) to species (tips of each branch). Node and edge (branch) width indicate the mean proportion of that taxon across all samples.


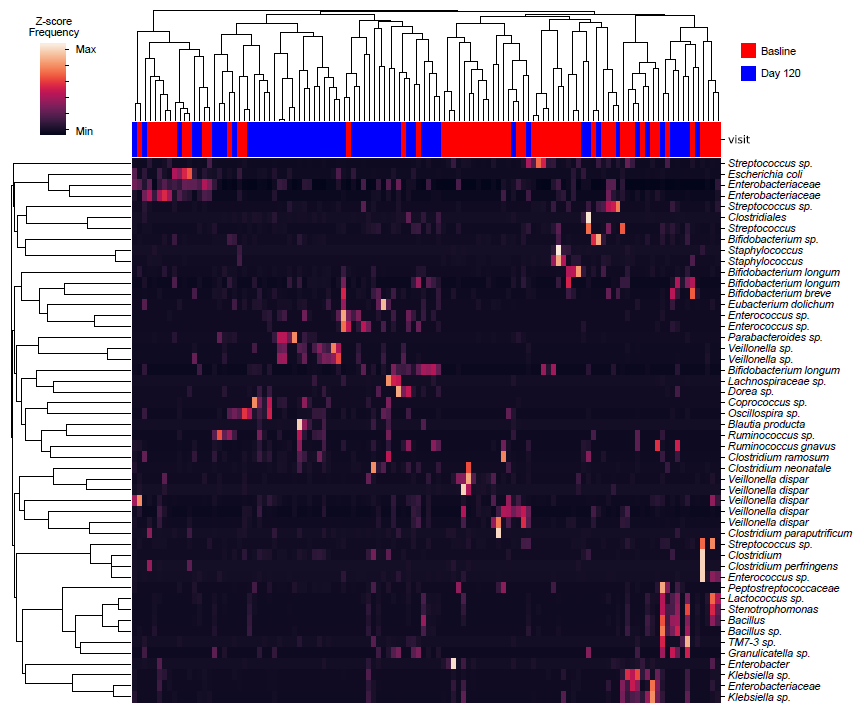

Supplement: nzab027_Supplemental_Files [file nzab027_supplemental_files.zip › Supplemental_Figures.docx]
