# Supplementary figures and images for: Effect of Bovine Milk Fat Globule Membrane and Lactoferrin in Infant Formula on Gut Microbiome and Metabolome at 4 Months of Age
Source: Curr Dev Nutr. 2021 Apr 2;5(5):nzab027. doi: 10.1093/cdn/nzab027 (PMC8105244; doi:10.1093/cdn/nzab027)

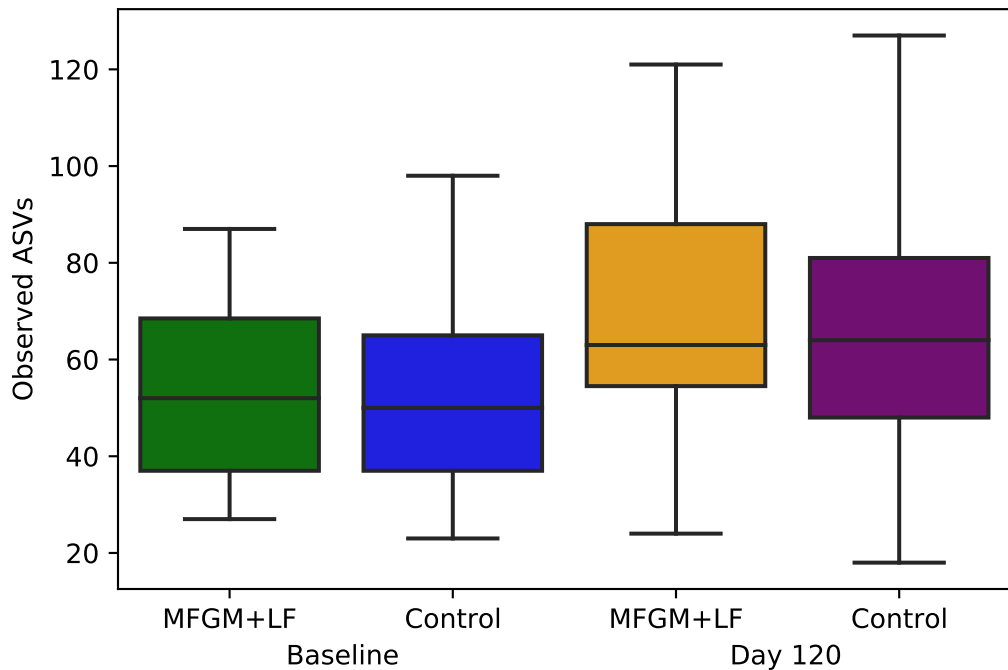

Supplement: nzab027_Supplemental_Files [file nzab027_supplemental_files.zip › Suppl_Fig_1_alpha_GroupVisit.pdf]

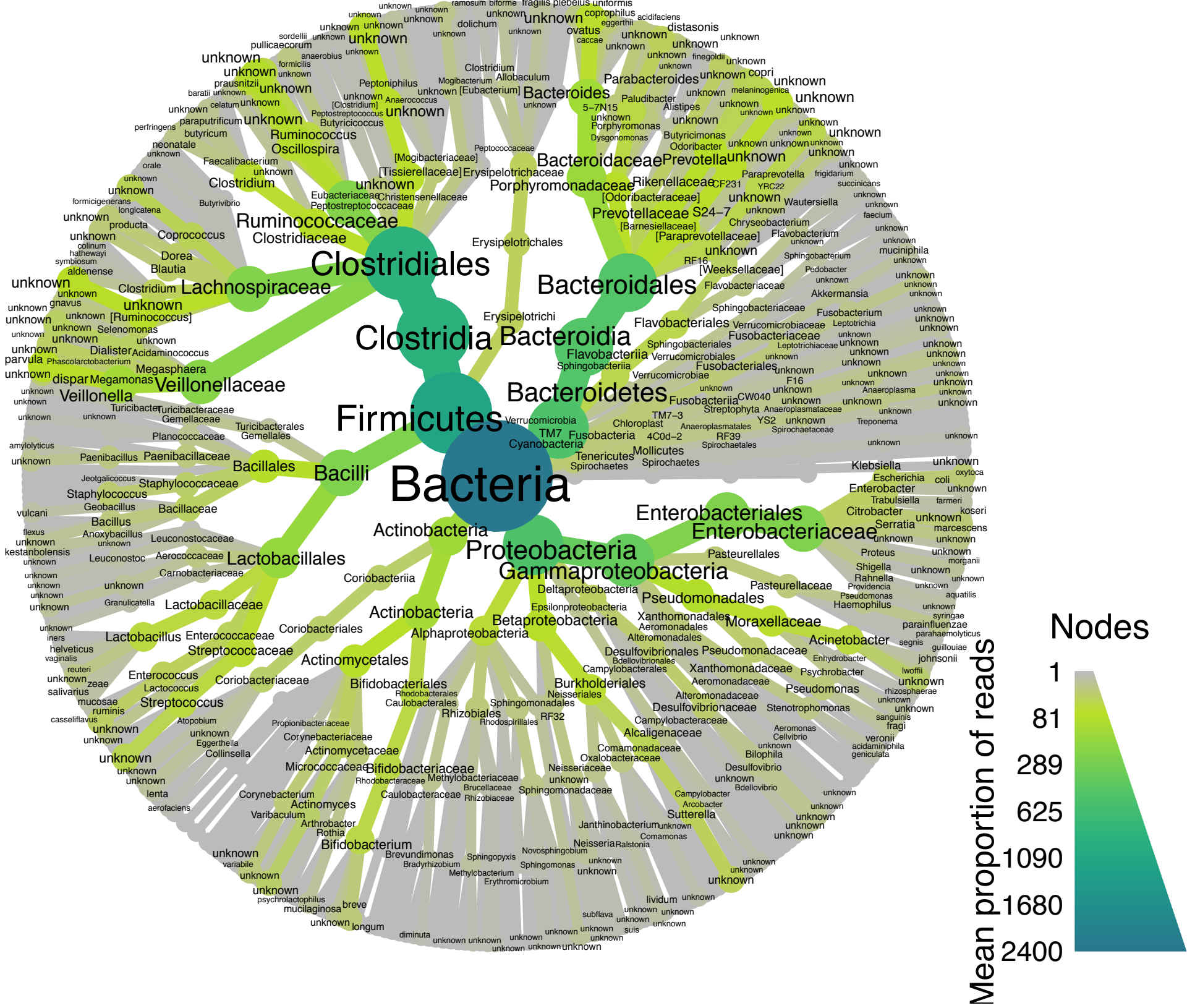

Supplement: nzab027_Supplemental_Files [file nzab027_supplemental_files.zip › Suppl_Fig_2_composition_heat_tree.pdf]

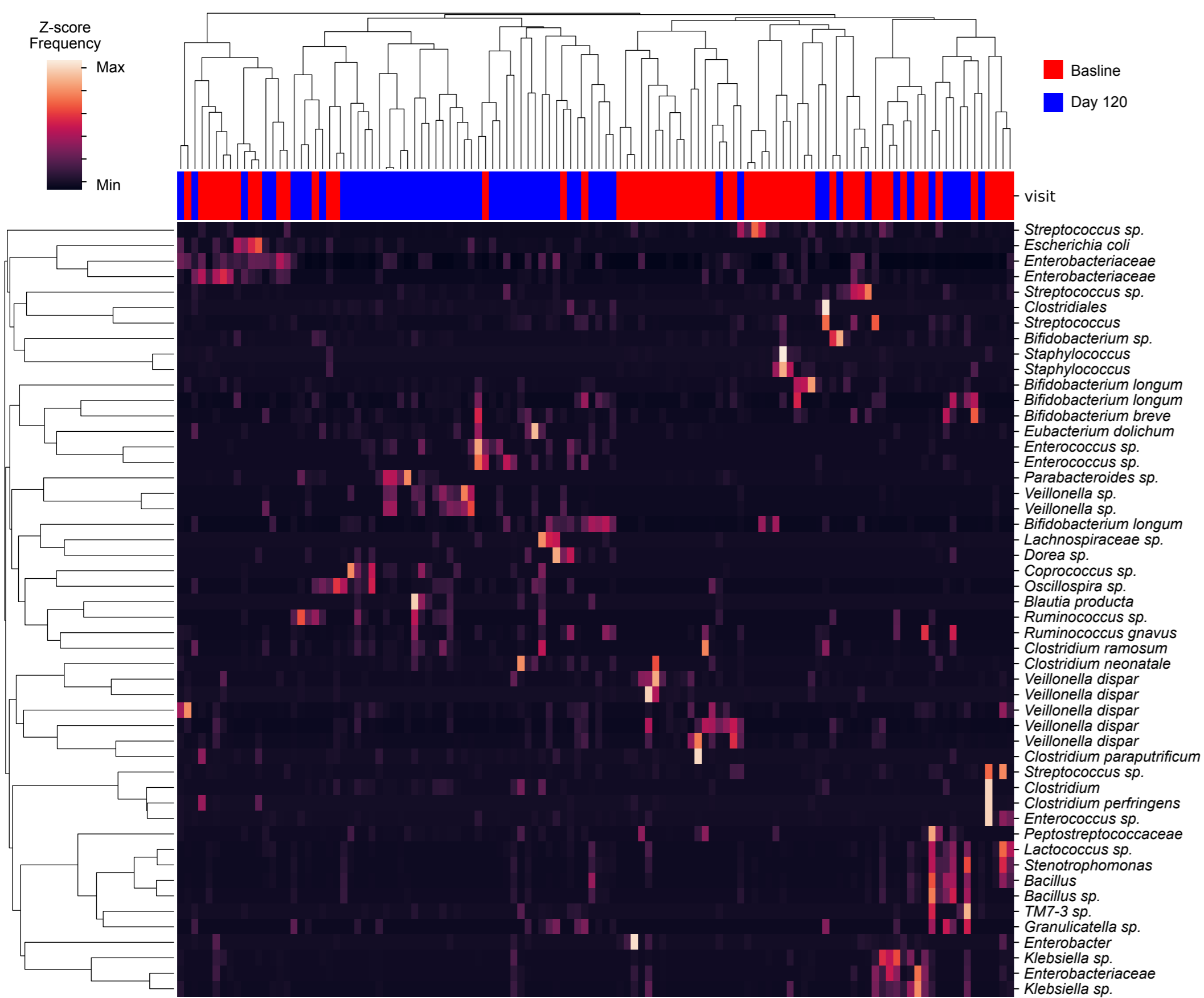

Supplement: nzab027_Supplemental_Files [file nzab027_supplemental_files.zip › Suppl_Fig_3_rf_importance_heatmap_final.pdf]
